# A Self-administered Version of the Functioning Assessment Short Test for Use in Population-based Studies: A Pilot Study

**DOI:** 10.2174/1745017902016010192

**Published:** 2020-09-25

**Authors:** Christoph Riegler, Silke Wiedmann, Viktoria Rücker, Henning Teismann, Klaus Berger, Stefan Störk, Eduard Vieta, Hermann Faller, Bernhard T Baune, Peter U Heuschmann

**Affiliations:** 1 Institute for Clinical Epidemiology und Biometry, University of Würzburg, Würzburg, Germany; 2 Department of Neurology, Charité - Universitätsmedizin Berlin (corporate member of Freie Universität Berlin, Humboldt-Universität zu Berlin), Berlin, Germany; 3 Charité - Universitätsmedizin Berlin, corporate member of Freie Universität Berlin, Humboldt-Universität zu Berlin, and Berlin Institute of Health; Berlin, Germany; 4 Institute for Epidemiology and Social Medicine, University of Münster, Münster, Germany; 5 Comprehensive Heart Failure Center, University and University Hospital Würzburg, Würzburg, Germany; 6 Department of Medicine I, University Hospital Würzburg, Würzburg, Germany; 7 Bipolar and Depressive Disorders Program, Institute of Neurosciences, Hospital Clinic, University of Barcelona, IDIBAPS, CIBERSAM, Barcelona, Spain; 8 Department of Medical Psychology and Psychotherapy, Medical Sociology and Rehabilitation Sciences, University of Würzburg, Würzburg, Germany; 9 Department of Psychiatry, Melbourne Medical School, The University of Melbourne, Melbourne, Australia; 10 Department of Psychiatry and Psychotherapy, University of Münster, Münster, Germany; 11 Clinical Trial Center Würzburg, University of Würzburg, Würzburg, Germany

**Keywords:** Functional Assessment Short Test, FAST, Population-based, Self-administered, German

## Abstract

**Background:**

The Functioning Assessment Short Test (FAST) is an interviewer-administered scale assessing functional impairment originally developed for psychiatric patients.

**Objectives:**

To adapt the FAST for the general population, we developed a self-administered version of the scale and assessed its properties in a pilot study.

**Methods:**

The original FAST scale was translated into German *via* forward and backward translation. Afterwards, we adjusted the scale for self-administered application and inquired participants from two ongoing studies in Germany, ‘STAAB’ (Würzburg) and ‘BiDirect’ (Münster), both recruiting subjects from the general population across a wide age range (STAAB: 30-79 years, BiDirect: 35-65 years). To assess reliability, agreement of self-assessment with proxy-assessment by partners was measured *via* intraclass correlation coefficient (ICC) over the FAST score. Construct validity was estimated by conducting correlations with validated scales of depression (PHQ-9), anxiety (GAD-7), and health-related quality of life (SF-12) and regression analyses using these scales besides potentially disabling comorbidities (*e.g.* Chronic Back Pain (CBP)).

**Results:**

Participants (n=54) had a median age of 57.0 years (quartiles: 49.8, 65.3), 46.3% were female. Reliability was moderate: ICC 0.50 (95% CI 0.46-0.54). The FAST score significantly correlated with PHQ-9, GAD-7, and the mental sub-scale of SF-12. In univariable linear regression, all three scales and chronic back pain explained variance of the FAST score. In multivariable analysis, only CBP and the SF-12 remained significant predictors.

**Conclusion:**

The German self-administered version of the FAST yielded moderate psychometric properties in this pilot study, indicating its applicability to assess functional impairment in the general population.

## 1 BACKGROUND

Assessment of Activities of Daily Living (ADL) is commonly used for measuring the individual and social consequences of clinical and subclinical diseases on patients’ functional capacity. In the setting of population-based studies, ADL questionnaires are essential tools to assess the individual and social consequences of clinical and subclinical diseases [1-3].

ADL can be further divided into basic ADL (BADL), *e.g.* eating/drinking, personal hygiene, or mobility, and instrumental ADL (IADL), *e.g.* financial issues, household, or intake of medication [4-6]. In the general population, most subjects manage their daily living independently and do not require assistance in BADL. Still, they might suffer from diagnosed as well as subthreshold conditions that can cause difficulties in IADL, *i.e.* depressive or anxiety symptoms [7-10], or unspecific syndromes such as back pain. There is only a limited number of IADL scales in the German-speaking area, and all available instruments were developed in a geriatric setting [6, 11-13]. Consequently, they lack questions on factors relevant for young and middle-aged subjects, among them occupational functioning, sporting activity, leisure time, and interpersonal relationships. In contrast, psychiatric IADL scales - such as the “Functioning Assessment Short Test” (FAST) - overcome this problem, since they were developed for patients of all ages. Such scales include questions on the above-mentioned areas as well as other IADL dimensions relevant also for younger age groups such as occupational functioning and sporting activity [14].

## 2 INTRODUCTION

The FAST was originally developed in Spain in 2007 to measure functional impairment among bipolar disorder patients [14]. The FAST score showed high internal consistency and test-retest reliability, and a good concurrent and discriminant validity [14]. Further, validation studies in Turkish, Italian, Brazilian Portuguese, Finnish, and Chinese confirmed the good psychometric properties of the FAST for bipolar disorder patients [15-20]. Additionally, the Spanish version of the FAST qualified as a useful instrument in obtaining functional impairment in patients with first psychotic episodes and attention deficit hyperactivity disorder [21, 22]. The Brazilian version was furthermore successfully validated in a sample of patients with schizophrenia [23] and patients with major depressive disorder [24]. The construction and content of the FAST scale are not specific to bipolar disorders. The functional components addressed are of general importance to humans, both with and without mental disorders. Hence, whereas the scale was developed as a clinician rated scale targeting bipolar disorders, it is not restricted to or specific for bipolar or even psychiatric disorders. Because of the general nature of the underlying psychosocial functional domains covered by the scale, we consider the scale applicable to healthy individuals as well as to disease groups.

The FAST has not been applied for functional assessment in population-based studies. This may be due to the fact that the FAST was constructed as an interview scale to be conducted by trained staff. As a result, its application is relatively time consuming, which constitutes a potential major barrier in large population-based studies.

Based on the FAST’s excellent psychometric properties, its coverage of all relevant IADL dimensions, and its applicability to subjects with a wide age range, we hypothesized that the FAST may be a useful instrument not only to assess psychiatric patients, but also subjects from the general population to measure IADL within the setting of population-based studies. To implement the FAST in population-based studies, we transformed the scale into a self-administered form aiming for a time-saving and efficient assessment of the participants’ functional capacities.

We report the results of developing a self-administered German version of the FAST adapted for use in the general population as well as of assessing its psychometric properties considering reliability and construct validity within a pilot study in two population-based samples in Germany.

## 3 METHODS

### 3.1 Development of the Self-administered Version of the Fast

The original version of the FAST is an ordinally scaled, interviewer-administered questionnaire containing 24 items out of six domains of functioning, *i.e.* autonomy, occupational functioning, cognitive functioning, financial issues, interpersonal relationships, and leisure time [14]. The English version (as provided in [14], see supplementary file 1) was translated into German by two native speakers following a standardized forward-backward translation protocol. To adjust the scale for the self-administered setting and the new target population (*i.e.* general population instead of psychiatric patients), some slight modifications of content were made to the original scale: a) Question 12 (regarding the ability to solve a problem adequately) was erased due to the following reason: In a self-administered questionnaire, cognitive functioning and the ability to solve problems relate to various areas of life. Therefore, this item is hard to operationalize and linguistically unspecific. Consequently, we expected a high variance due to inaccurate responses and chose to erase the item; b) question 21 (regarding a satisfactory sexual relationship) was modified to regard the satisfactory *emotional* relationship with the partner, since the hypersexual component of bipolar disorder is less relevant in a population-based sample. Besides, we considered the question regarding a satisfactory sexual relationship with the partner as too offensive in a study assessing the general population and in the setting of a self-administered questionnaire.

A text instructing the participants on how to fill in the questionnaire was added (see supplementary file 2). The option ‘*Not applicable for me*’ was added to the original ordinal scale, which contains the selection options ‘*No difficulty*’, ‘*Slight difficulty*’, *’Moderate difficulty*’ and ‘*Severe difficulty*’. A proxy version of the questionnaire was developed, allowing cohabitating partners to evaluate the subjects’ functional performance (proxy assessment; see supplementary file 3). This version additionally contained the selection option *‘Cannot be judged by me’*.

To obtain an overall rating of everyday functioning, we added two more questions to the questionnaires: first, an ordinally scaled question asking for ‘*General difficulties while managing daily activities’* with the above-mentioned selection options. Second, a Visual Analogue Scale (VAS) that had to be answered at the end of the questionnaire, reaching from 0 (*i.e.*, *problems with all mentioned IADL tasks*) to 100 (*i.e.*, *no problems with the mentioned IADL tasks*). Finally, the original time frame of 15 days preceding the examination was expanded to cover a total of 28 days, since we assumed substantial intra-individual variation of everyday functioning over time and expected a better regression to the mean using a larger time frame.

### 3.2 Assessment of Reliability

The reliability of the self-administered version of the FAST was examined by measuring the agreement of self-assessment with proxy assessment. For this purpose, all study subjects a) had to rate themselves, and b) had to be rated by their partners. The cohabitation of subjects and partners was obligatory to ensure that all proxies had close insights into their partner’s abilities to perform IADL. Cronbach’s alpha was calculated to assess the questionnaire’s internal consistency.

### 3.3 Assessment of Validity

The questionnaire’s construct validity was assessed by calculating correlations of the FAST score with well-known validated scores of depression (PHQ-9), anxiety (GAD-7), and health-related quality of life (SF-12). Furthermore, predictors of higher FAST scores (indicating more difficulties in IADL) were identified by conducting linear regression analyses using the above-mentioned scales besides potentially disabling comorbidities (depression, arthrosis, chronic back pain, heart failure, stroke) obtained from the subjects’ medical record.

### 3.4 Subject Recruitment

The study sample was derived from two ongoing population-based studies in Germany: the STAAB study in Würzburg aims “to determine the prevalence of heart failure stages A-B in a representative sample of the general population and to prospectively investigate the progression from asymptomatic cardiac dysfunction into symptomatic heart failure” [25]. In the BiDirect study in Münster, ”the bidirectional relationship between depression and (subclinical) atherosclerosis” is explored [26]. Both studies recruited subjects *via* the local residents registration offices with a wide range of age at enrolment (Würzburg: ≥30 to <80 years, Münster: ≥35 to <66 years). From each study, we recruited 30 study participants as well as their cohabitating partners.

### 3.5 Data Acquisition

All subjects from Würzburg were visited in their homes by a member of the study staff, which ensured that the participants and their partners did not communicate while filling out the FAST questionnaires. In Münster, all subjects were invited to the study centre of the BiDirect study to answer the questionnaire under similar conditions, *i.e.* under supervision. Whenever the participants and their partners could not visit, they were sent separate prepaid envelopes containing the questionnaires, which had to be resent separately to the study centre after completion. In both studies, the variables administered to assess construct validity were derived from the last preceding visit of the participants at the study site.

### 3.6 Statistical Analyses

By adding up the 23 ordinally scaled items with four valid categories (‘*No difficulty’*=0 points, ‘*Slight difficulty’*=1 point, ‘*Moderate difficulty’*=2 points, ‘*Severe difficulty’*=3 points), the FAST score comprising a range from 0 to 69 was calculated. According to a priori defined criteria, pairs of raters answering more than four items with ‘*Not applicable for me’* or ‘*Cannot be judged by me’,* respectively, were excluded from the calculation of the sum score. If the participant was retired or not working, seven ‘missing items’ were allowed. In all other cases, the valid items (‘no/slight/moderate/severe difficulty’) were summed up, their means were calculated, and then multiplied by 23 to obtain the sum score. Hence, the invalid items answered ‘Not applicable for me’ or ‘Cannot be judged by me’ were replaced by the mean of all valid items of the participant. Agreement of self- and proxy assessment of the FAST score was assessed by calculating intraclass correlation coefficients (ICCs). Considering the not normally distributed data, a non-parametric, rank-based approach was chosen [27, 28]. To assess a possible influence of the mode of inquiry (under supervision *vs.* unsupervised - see ‘data acquisition’), sensitivity analyses with the calculation of separate ICCs for both groups were carried out. Agreement at single item-level was measured *via* weighted kappa with radical weights, following the method of Brennan and Prediger [29, 30]. The agreement of the VAS was assessed *via* the above-mentioned non-parametric ICC. To assess construct validity, spearman-correlations with the SF-12, PHQ-9, and GAD-7 scores were calculated. Additionally, regression analyses with these scales and variables from the subjects’ medical record (potentially disabling comorbidities) as independent variables and the FAST sum score (self-assessment) as the dependent variable were conducted. SF-12, PHQ-9, GAD-7 and age (in decades) were entered as continuous variables, whereas comorbid diagnoses were entered as dichotomous variables. The FAST sum scores were transformed with [ln (1+ sum score FAST)] to make them applicable for linear regression. After univariable regression, a stepwise backward multivariable regression was performed. Analogous correlations and regressions were computed to identify predictors for the deviation between self- and proxy assessment regarding the FAST sum score.

A power calculation was conducted prior to recruitment. It was assumed to detect an ICC of 0.8 at a power of 90% with a sample size of 30 participants, which was the aimed sample size in Würzburg and Münster, respectively.

The correlation and regression analyses were conducted with SPSS, version 25. The non-parametric ICC was calculated with MS Excel, version 15.3, and the weighted kappa coefficients were calculated with R, version 3.4.2.

## 4 RESULTS

### 4.1 Recruitment and Basic Demographic Characteristics

Recruitment and data acquisition took place between December 2015 and November 2016 in Münster, and between March and June 2016 in Würzburg. A total population of 60 individuals was examined (30 from Würzburg, and 30 from Münster). Four subjects of the Münster cohort were excluded, since they were erroneously not recruited from the general population, but were part of the BiDirect study cohort of patients with depression, which had been recruited from psychiatric departments. Two pairs of raters were excluded from FAST sum score calculation because too many items had been answered outside the predefined boundaries. Thus, 54 subjects remained for analysis of the sum score, while 56 subjects were suitable for analysis of agreement at single-item level. Of the former, 44 (81.5%) answered the questionnaire under supervision, while 10 (18.5%) were inquired by mail, *i.e.* unsupervised.

Since the subjects from Würzburg and Münster were derived from two comparable population-based cohort studies and had similar median values of the FAST sum score, a pooled analysis was conducted. Table **1** illustrates the basic demographic characteristic for the two individual samples as well as for the pooled sample. Furthermore, it depicts information on the participants’ working status and the number of items answered inside the valid categories. The pooled study population included subjects from a wide age range (33-71 years) and had nearly balanced proportions of sex. The general level of difficulties with IADL was quite low, and most participants had long-term relationships with the partners that performed proxy assessment. While the employed participants answered a median of 22 items (quartiles 21, 22) inside the valid categories, the retired participants answered only a median of 19 items (18, 19), respectively.

### 4.2 Reliability

With a Cronbach’s alpha of 0.76, the self-administered version of the FAST showed acceptable internal consistency. The agreement of self-assessment with a proxy assessment regarding the sum score was moderate: ICC 0.50 (95%-confidence interval (CI) 0.46-0.54). Fig. (**1**) illustrates the distribution of sum scores and the deviation from perfect agreement for each pair of raters. Since 18.5% of the participants answered the questionnaire unsupervised, it seems possible that these subjects and their partners did not answer the questionnaires independently. However, after excluding the unsupervised pairs of raters, the agreement for the FAST sum score differed only slightly: ICC 0.53 (95% CI 0.49-0.58). The excluded pairs of raters were analysed separately; the results showed poor agreement: ICC 0.38 (95% CI 0.11-0.64).

With a mean weighted kappa coefficient of 0.63 (95% CI 0.57-0.69), the agreement on single-item level was moderate, too. Table **2** shows weighted kappa coefficients for all ordinally scaled items. Weight matrices are depicted in Table **3**.

Regarding the VAS, the agreement was poor: ICC 0.32 (95% CI 0.28-0.35). Fig. (**2**) shows the distribution of VAS scores and the deviation from the perfect agreement.

### 4.3 Validity

In both studies of Würzburg and Münster, well-adopted and validated scales of depression, anxiety, and health-related quality of life were obtained. However, the information was not sampled simultaneously with the FAST questionnaire, but at the subjects’ last regular visit at the study centres (*i.e.*, baseline visit in Würzburg, second follow-up visit in Münster). While most of the participants from Würzburg were assessed with the FAST shortly after their baseline visit, the second follow-up visit of the subjects from Muenster took place more than two years before their FAST assessment. Therefore, construct validity was only analysed for the subgroup of participants from Würzburg. Three of these subjects were excluded, because their baseline visit preceded the FAST assessment by more than one year. For the remaining 27 participants, the median interval between STAAB baseline visit and FAST assessment was 6 days (IQR 5-8 days). As detailed in Table **4**, significant correlations between the participants’ FAST score and the PQH-9 and GAD-7 scores were found. Thus, higher difficulties in performing IADL were found in the presence of increased symptoms of depression and anxiety. Further, a significant correlation between the participants’ FAST sum score and the mental sub-scale of the SF-12 was found. Participants with a lower level of mental health-related quality of life thus reported higher difficulties in IADL. In univariable linear regression analyses, a diagnosis of heart failure, myocardial infarction, stroke, diabetes mellitus, arthrosis and depression at any time in the participant's medical record had no significant influence on the FAST score of the participants. In contrast, chronic back pain, PHQ-9, GAD-7, and the mental sub-scale of the SF-12 significantly influenced the FAST sum score in univariable models. However, only chronic back pain and the SF-12 mental sub-scale remained significant in multivariable analysis (R^2^ of the final model: 0.53, corrected R^2^: 0.48). For point estimators and p-values see Table **5** (results for clearly non-significant comorbidities, *i.e.* heart failure, myocardial infarction, diabetes mellitus, stroke, and arthrosis, are not shown).

### 4.4 Predictors of Deviation between Self and Proxy-assessment

Regarding the absolute value of deviation of self-assessment and proxy assessment in the FAST sum score, there was only a significant positive correlation with the PHQ-9 score: higher deviation (without regarding its direction) prevailed in participants that reported more severe symptoms of depression. Table **6** contains the exact values and further correlations. In univariable linear regression analyses, the PHQ-9 (p=0.08) as well as the GAD-7 (p=0.07) turned out to be tendentially significant predictors, while the SF-12 physical sub-scale had a significant influence on the absolute value of deviation: point estimate 0.79 (95% CI 0.64-0.97); p=0.03). A higher deviation was thus present in participants with lower physical health-related quality of life. Details are shown in the lower part of Table **6**. No significant correlations were found when the direction of deviation was considered.

## 5 DISCUSSION

### 5.1 Integration of Results into Previously Published Articles

The newly-developed, self-administered version of the FAST showed acceptable internal consistency, good construct validity, and a moderate to poor reliability in two population-based studies.

The median (quartiles) score of the FAST was 6.33 (2.27, 12.48); this value is comparable to the healthy control groups in the validation studies of Rosa in Spain (mean 6.07, SD: 4.72) [14], Barbato in Italy (mean 7.90, SD 11.44) [19], Zortéa in Brazil (median 5.0) [23], and Rotger in Spain (mean 6.01, SD 4.67) [21].

Due to the self-administered application of the scale, reliability could not be assessed *via* interrater reliability. Instead, we chose to assess reliability by assessing the agreement of self-assessment with proxy assessment by partners. We chose this approach for two reasons: First, there is no comparable German IADL scale validated for the general population that could be used as a reference standard. Second, this is the first German version of the FAST. Since we aimed to use the scale in population-based studies, we did not develop an interviewer-administered German version of the FAST that could have been used to measure reliability by testing agreement of self-assessment with interviewer-assessment.

So far, no studies investigating the agreement of self-assessment with a proxy assessment of the FAST have been published. Therefore, our results cannot be compared directly. Ostbye *et al.* reported a comparable, only moderate level of agreement (weighted kappa 0.55; 95% CI 0.48-0.62) in an elderly, non-demented cohort when comparing ADL and IADL self-assessment with proxy assessment by caregivers [31]. However, in this study, the examined population was older, and the group of caregivers consisted not only of partners but also of offspring and others, which limits the comparability to our population.

The moderate agreement regarding the sum score of the FAST should not inevitably be interpreted as a sign of poor quality of the instrument itself. Multiple factors might have influenced how the participants and their partners judged the participants’ functional status. For instance, higher deviations were found when the participants reported symptoms of depression and anxiety and a lower physical health-related quality of life. These or other undocumented factors may also prevail in the partners and could have influenced their judgement of the participants’ functional status, which may explain part of the deviation. Zanetti *et al.* compared caregiver’s proxy assessment with direct performance-based assessment of functional impairment in patients with very mild or mild dementia and reported that the agreement was influenced by the caregivers’ burden [32]. This indicates that internal factors of proxies performing IADL assessment can affect their rating. However, since no further variables of the partners were obtained, it is not possible to confirm this hypothesis in our data.

Since there may be substantial bias of both over- and underestimation in the participants’ as well as their partners’ judgements, we would recommend using methods beyond the agreement of self-assessment and proxy assessment in further validation studies.

Considering the agreement on single-item level, it was notable that the concordance for tasks that could be directly observed was higher than for items that comprised more abstract or subjective tasks. As an example, the agreement about difficulties while ‘*taking care of oneself (physical aspects, hygiene)*’ was much higher than for difficulties while ‘*concentrating on a book or film’*. With a mean weighted kappa coefficient of 0.63 (95%-CI: 0.57 - 0.69) and a Cronbach’s alpha of 0.76, we judge the reliability on single item level as satisfactory and thus see no need to eliminate items. However, it should be noted that the Cronbach’s alpha of our German version of the FAST is considerably lower compared to the previously published FAST studies, which reached values from 0.87-0.96 [14-23].

**Fig. (2):**
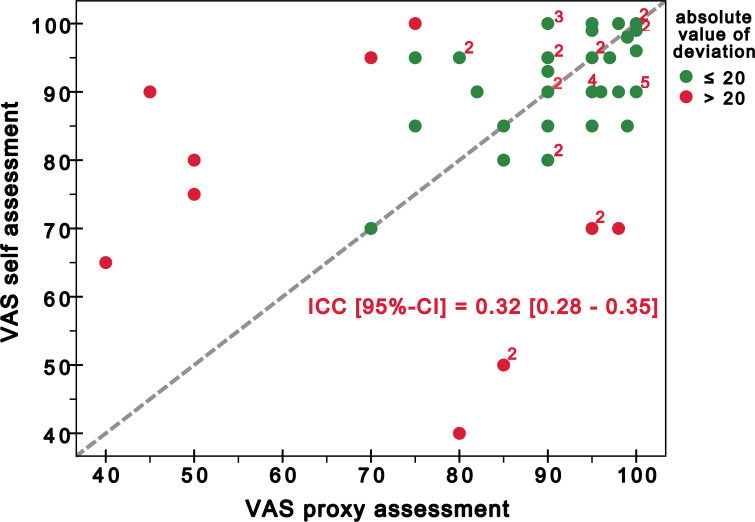
Scatterplot of the VAS scores for self-assessment and proxy assessment. Higher values indicate fewer difficulties while performing IADL. The dashed line symbolizes perfect agreement. Highlighted numbers are used whenever more than one pair of raters had identical results (*e.g.* a 2 illustrates that the dot to which it is attached symbolises two pairs of raters with identical results).

The VAS used in the self-administered version of the FAST reflected poor agreement of self-assessment with proxy assessment. This is on the one hand, due to the many outliers; on the other hand, this is due to the high occurrence of shared ranks (see the superscript numbers in Fig. **2**) that influence the rank-based approach more than if a parametric method had been used. Since the VAS is highly negatively correlated with the FAST sum score (ρ=-0.78, p≤0.001), we consider it as a dispensable element carrying no additional information and propose to omit it when applying the questionnaire in clinical studies.

The significant correlations of the FAST sum score with PHQ-9, GAD-7, and the mental sub-scale of the SF-12 are consistent with numerous previous findings: in elderly patients, there is evidence for an association between symptoms of depression or anxiety and IADL impairment [7-9, 33]. Additionally, functional impairment has been reported in adult subjects with sub-threshold symptoms of depression and anxiety disorder [10, 34, 35]. Furthermore, poor mental health-related quality of life was associated with functional-impairment in two geriatric studies [36, 37]. The fact that those associations were reproduced in our study points towards a high construct validity of the German version of the FAST. Whilst the psychiatric scales significantly influenced the FAST score, most somatic comorbidities (as heart failure, stroke or arthrosis) did not influence the FAST score. We hypothesize this to be due to 1) the low prevalence of these diseases in the study sample and 2) the fact that participants were asked for a diagnosis at any point in medical history (regardless of actual disabilities as a consequence).

### 5.2 Limitations and Strengths

Since the ICC of 0.5 observed in our study was considerably lower than the anticipated ICC of 0.8 that had been used for sample size calculation, the study can be considered underpowered. Furthermore, a power calculation was performed only for the primary analysis, *i.e.* the detection of the ICC. As the further statistical analyses were only explorative secondary analysis, we did not adjust the p-value for multiple testing. Hence, all further results need to be interpreted with caution. We did not apply a qualitative approach (*e.g.* focus groups) to ensure that all questions were understood adequately by all participants before beginning the validation process of the scale. This might have added to the rather low agreement between self- and proxy-assessment. We did not assess test-retest reliability since we assumed substantial intra-individual variance of the FAST score over time and concluded this would bias the results. However, we may have been able to assess test-retest reliability by choosing a rather short time gap between the two points of inquiry. Furthermore, we did not assess concurrent validity of the FAST with already validated IADL scales since there is no comparable scale in German language containing all relevant IADL dimensions that have been validated in samples of the general population and could be used as a reference standard.

Still, the correlations and regression analyses with the above mentioned validated scales illustrate the high construct validity of the German version of the FAST. Yet, it has to be noted that the FAST score and the other variables utilized to evaluate construct validity were not gathered simultaneously. However, the time interval between the two points of inquiry was not longer than one week for most of the participants, leading to a substantial overlap of the timeframes of the observation periods covered per scale. Additionally, it is reasonable to assume that health-related quality of life and symptoms of depression and anxiety do not change substantially within only a few days. We did not perform a factorial analysis due to the small sample size. However, given that only very few items were modified, and given that the original factorial structure has been reproduced in other validation studies of the instrument [18-20], it can be supposed that the factorial structure of the German version is similar to the original one. As 18.5% of the pairs of raters conducted the FAST assessment unsupervised, some of them may have answered the questionnaires not independently. However, the raters that were inquired unsupervised had only poor agreement when being analysed separately. Hence, it seems unlikely that they agreed on results. Although this study examined participants of a broad age range, younger (30-40 years) and elderly (>70 years) subjects are underrepresented in our sample. The results are therefore restricted to middle-aged to early late-aged adults. The generalizability of this study is further limited, since only participants with a cohabitating partner were recruited.

The comparability of our results regarding the self-administered version of the FAST with the previously published FAST studies is limited due to the following reasons: a) while the previous studies were carried out in samples of psychiatric patients, we tested the FAST in a sample derived from the general population, and b) we performed some slight modifications of content to adjust the scale for the new target population and self-administered assessment as detailed in the methods section.

This is the first study evaluating a self-administered version of the FAST. The scale’s coherence with well-adopted, validated scales of depression and anxiety can be considered a new and valuable finding since it illustrates the FAST’s applicability in measuring the impact of even slight manifestations of these symptoms on everyday functioning. A methodological strength of this study is the fact that a non-parametric ICC approach and Spearman correlations were applied, thereby accounting for the distributional structure of the data, instead of simply using conventional parametric methods.

## CONCLUSION

In this pilot study, the self-administered version of the FAST showed good construct validity, but only moderate to poor reliability in subjects from the general population in Germany. However, we assume that these results are mainly due to the method of assessment of reliability (agreement of self-assessment with proxy assessment by partners) and do not point towards poor quality of the instrument itself. For the further validation process of the German version of the FAST, we suggest developing and validating an interviewer-reported German version of the FAST as a next step. If this version will yield good psychometric properties, it could be used as a reference standard in another approach to validate a self-administered German version of the FAST by comparing self-assessment with interview-assessment in a larger and more balanced sample.

## Figures and Tables

**Fig. (1):**
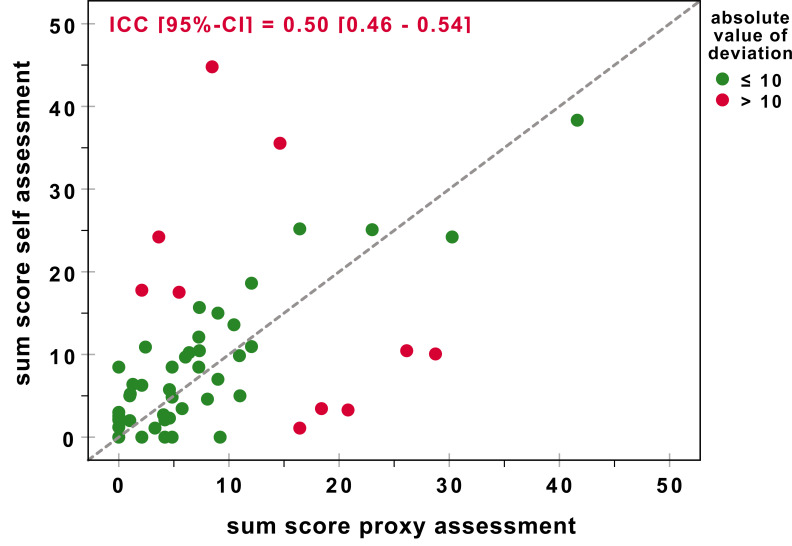
Scatterplot of the FAST sum scores for self-assessment and proxy assessment Higher values indicate more difficulties while performing IADL. The dashed line symbolises perfect agreement.

**Table 1:** Demographic characteristics and median FAST sum scores.

	**Pooled data ** **(n=54)**	**Würzburg ** **(n=30)**	**Münster ** **(n=24)**			
Age (years) - Median (quartiles)	57.0 (49.8, 65.3)	54.5 (48.8, 64.0)	59.0 (51.0, 67.0)			
Female sex - N (%)	25 (46.3)	14 (46.7)	11 (45.8)			
FAST sum score^1^ - Median (quartiles)	6.3 (2.3, 12.5)	7.7 (2.9, 12.8)	5.5 (2.1, 12.9)			
Employed - N (%)	35 (64.8%)	18 (60.0%)	17 (70.8%)			
Number of items answered inside the valid categories^2^ - Median (quartiles)	21 (19, 22)	21 (19, 22)	21 (19, 22)			
Duration of partnership (years) - Median (quartiles)	34.5 (19.8, 45.3)	30.0 (17.0, 43.5)	36.0 (29.3, 46.8)			

^1^ Higher values indicate more difficulties while performing IADL.

^2^ Valid categories: ‘No difficulty’, ‘Slight difficulty’, ‘Moderate difficulty’, ‘Severe difficulty’. Invalid categories: ‘Not applicable for me’, ‘Cannot be judged by me’.

**Table 2:** Weighted kappa coefficients for ordinally scaled items.

**Item**	**Directly observable**	**Did you experience difficulties …?**	**Weighted kappa** **[95% CI]**	**Item**	**Directly observable**	**Did you experience difficulties …?**	**Weighted kappa** **[95% CI]**
4		‘in general’	0.59 [0.44-0.74]	5.12		‘remembering newly-learned names’	0.25 [0.10-0.41]
5.1	X	‘taking responsibility for a household’	0.69 [0.54-0.83]	5.13		‘learning new information’	0.49 [0.33-0.66]
5.2		‘living alone’	0.73 [0.34-1.00]*	5.14	X	‘managing your own money’	0.77 [0.64-0.92]
5.3	X	‘doing the shopping’	0.82 [0.69-0.94]	5.15	X	‘spending money in a balanced way’	0.76 [0.63-0.90]
5.4	X	‘taking care of yourself (physical aspects, hygiene)’	0.85 [0.73-0.97]	5.16		‘maintaining a friendship or friendships’	0.48 [0.29-0.67]**
5.5	X	‘holding down a paid job’	0.82 [0.66-0.98]	5.17		‘participating in social activities’	0.52 [0.35-0.69]
5.6	X	‘accomplishing tasks as quickly as necessary’	0.65 [0.50-0.81]	5.18	X	‘having good relationships with people close to you’	0.66 [0.52-0.80]
5.7		‘working in the field you were educated‘	0.66 [0.45-0.87]	5.19	X	‘living together with your family’	0.72 [0.59-0.85]
5.8		‘earning a sufficient wage’	0.88 [0.75-1.00]**	5.20		‘having an emotionally satisfactory relationship’	0.57 [0.42-0.72]
5.9		‘managing the expected work load’	0.58 [0.43-0.74]	5.21		‘being able to defend your interests’	0.51 [0.34-0.68]
5.10		‘concentrating on a book or film’	0.49 [0.33-0.66]	5.22	X	‘doing exercise or participating in sport’	0.45 [0.27-0.62]
5.11		‘while making mental calculations’	0.53 [0.37-0.69]	5.23		‘having hobbies or personal interests’	0.62 [0.46-0.78]

For the unmarked items, all 4 categories were used by the raters. One item marked * had only two used categories (unweighted kappa was calculated). Items marked ** had 3 used categories. The weight matrices changed with the number of used categories (see Table **3A** and **B**). The item numbers in the table refer to the German scale (see supplementary files 2 and 3). Regarding item 5.1 to item 5.11, the order of items is identical to the original English version of the scale (see supplementary file 1, *e.g.* item 1 in the original scale became item 5.1 in the German scale). Since item 12 of the original English scale is not present in the self-administered German version, the number of the items 5.12 to 5.23 of the German scale is one point lower than the corresponding item in the original English scale (*e.g.* item 13 of the English scale corresponds to item 5.12 in the self-administered German scale).

**Table 3:** Weight matrices for weighted kappas.

**A - Weight matrix for items with all four categories used by the raters**						
	**No difficulties**	**Slight difficulties**		**Moderate difficulties**		**Severe difficulties**
No difficulties	1	0.42		0.18		0
Slight difficulties	-	1		0.42		0.18
Moderate difficulties	-	-		1		0.42
Severe difficulties	-	-		-		1
**B - Weight matrix for items with three categories used by the raters**						
-	**No difficulties**		**Slight difficulties**		**Moderate difficulties**	
No difficulties	1		0.29		0	
Slight difficulties	-		1		0.29	
Moderate difficulties	-		-		1	

**Table 4:** Correlations of the FAST sum score*^1^ with validated scales.

-	**Spearman’s rho**	**p**		
PHQ-9 - Depression symptom severity^1^	0.57	<0.01		
GAD-7 - Fear and anxiety^1^	0.50	<0.01		
SF-12 - Physical sub-scale^2^	-0.01	0.95		
SF-12 - Mental sub-scale^2^	-0.70	<0.001		

* Self-assessment

^1^ Higher values on the scale indicate a higher burden of depressive symptoms (PHQ-9), symptoms of anxiety (GAD-7), or functional impairment (FAST).

^2^ Higher values on the scale indicate better health-related quality of life.

**Table 5:** Variables potentially affecting the FAST sum score.

	**Univariable regression ** **coefficient [95% CI]**	**p**
Age (per +10 years)	0.86 [0.60-1.25]	0.42
Female sex	1.26 [0.61-2.58]	0.52
Depression^2^	1.81 [0.67-4.93]	0.23
Chronic back pain^2^	2.58 [1.19-5.59]	0.02
PHQ-9 (per +1 point)^1^	1.12 [1.02-1.21]	0.02
GAD-7 (per +1 point)^1^	1.21 [1.08-1.37]	<0.01
SF-12 physical sub-scale^3^ (per +5 points^4^)	0.91 [0.72-1.16]	0.45
SF-12 mental sub-scale^3^ (per +5 points^4^)	0.74 [0.63-0.87]	<0.01
-	**Stepwise backwards ** **regression coefficient [95% CI]**	**p**
SF-12 mental sub-scale^2^ (per +5 points)	0.76 [0.66-0.88]	<0.01
Chronic back pain^3^	2.30 [1.17-4.54]	0.02
*Stepwise excluded variables: PHQ-9 (p=0.68), GAD-7 (p=0.72)* **R^2^ for final model 0.53, corrected R^2^ 0.48**		

Data derived from regression analysis with (ln (1 + sum score self-assessed FAST) as dependent variable.

^1^ Higher values on the scale indicate a higher burden of symptoms of depression (PHQ-9), anxiety (GAD-7), or more difficulties in performing instrumental activities of daily living (FAST).

^2^ Recorded at any time in the medical history of the participant.

^3^ Higher values on the scale indicate the better health-related quality of life

^4^ Since the SF-12 score has a wide range, we chose steps of 5 points to calculate regression coefficients.

**Table 6:** Factors explaining the deviation between self-assessment and proxy assessment.

A - Correlations of the modulus of deviation* of the FAST sum score^1^ **with validated scales**		
-	**Spearman’s rho**	**p**
PHQ-9 - depression symptom severity^1^	0.41	0.04
GAD-7 - fear and anxiety^1^	0.16	0.43
SF-12 - physical sub-scale^2^	-0.16	0.42
SF-12 - mental sub-scale^2^	-0.22	0.28
B - Univariable regression on [ ln (1 + (modulus of deviation* of the FAST sum score^1^) ]		
-	Regression coefficient [95%-CI]	p
PHQ-9 (per +1 point)^1^	1.08 [0.99-1.17]	0.08
GAD-7 (per +1 point)^1^	1.12 [0.99-1.28]	0.07
SF-12 physical sub-scale^2^ (per +5 points)	0.79 [0.64-0.97]	0.03
SF-12 mental sub-scale^2^ (per +5 points)	0.88 [0.73-1.06]	0.17

* Self-assessment *vs.* proxy-assessment

^1^ Higher values on the scale indicate a higher burden of symptoms of depression (PHQ-9), of anxiety

(GAD-7), or more difficulties in performing instrumental activities of daily living (FAST).

^2^ Higher values implicate better health-related quality of life.

## LIST OF ABBREVIATIONS

FAST: = Functioning Assessment Short Test
ADL: = Activities of Daily Living
BADL: = Basic Activities of Daily Living
IADL: = Instrumental Activities of Daily Living
STAAB: = Stadium A and B (of Heart Failure) – population-based cohort study in Würzburg from which half of the studied participants were derived.
VAS: = Visual Analogue Scale
ICC: = Intraclass Correlation Coefficient
SF-12: = Short Form 12
PHQ-9: = Patient Health Questionnaire 9-item depression scale
GAD-7: = 7-item Generalized Anxiety Disorder scale
CI: = Confidence Interval
